# Low Frequency Vibrations Induce Malformations in Two Aquatic Species in a Frequency-, Waveform-, and Direction-Specific Manner

**DOI:** 10.1371/journal.pone.0051473

**Published:** 2012-12-10

**Authors:** Laura N. Vandenberg, Claire Stevenson, Michael Levin

**Affiliations:** Biology Department, Center for Regenerative and Developmental Biology, Tufts University, Medford, Massachusetts, United States of America; Deakin School of Medicine, Australia

## Abstract

Environmental toxicants such as industrial wastes, air particulates from machinery and transportation vehicles, and pesticide run-offs, as well as many chemicals, have been widely studied for their effects on human and wildlife populations. Yet other potentially harmful environmental pollutants such as electromagnetic pulses, noise and vibrations have remained incompletely understood. Because developing embryos undergo complex morphological changes that can be affected detrimentally by alterations in physical forces, they may be particularly susceptible to exposure to these types of pollutants. We investigated the effects of low frequency vibrations on early embryonic development of two aquatic species, *Xenopus laevis* (frogs) and *Danio rerio* (zebrafish), specifically focusing on the effects of varying frequencies, waveforms, and applied direction. We observed treatment-specific effects on the incidence of neural tube defects, left-right patterning defects and abnormal tail morphogenesis in *Xenopus* tadpoles. Additionally, we found that low frequency vibrations altered left-right patterning and tail morphogenesis, but did not induce neural tube defects, in zebrafish. The results of this study support the conclusion that low frequency vibrations are toxic to aquatic vertebrates, with detrimental effects observed in two important model species with very different embryonic architectures.

## Introduction

For several decades, the field of environmental toxicology has been expanding its focus from identifying toxicants and determining their sources of exposure, to assessing the effects of these toxicants on target and non-target species, and determining their mechanisms of action. A large amount of attention has been given to chemicals found in the environment that may be affecting wildlife species including mutagens, carcinogens, and reproductive/developmental toxicants; some of these chemicals have been proposed as contributors to the decline in amphibian populations that has been observed [1], [2]. However, there are other environmental perturbations that have received less attention including the effects of noise, electromagnetic fields, and low frequency vibrations. Should these environmental factors be considered developmental toxicants?

In many species, the period encompassing organogenesis is most susceptible to disruption by environmental toxicants because alterations in developmental processes during these stages can have permanent results [3]. Embryogenesis is marked by many complex morphological changes, regardless of whether embryos develop externally (i.e. fish, frogs), *in ovo* (i.e. chicks, reptiles), or *in utero* (i.e. mammals) [4], [5], [6], [7]. We propose that because low frequency vibrations can disrupt the cytoskeleton of treated cells [8], [9], they alter morphogenesis of the developing embryo and are therefore toxic. We previously examined the effects of low frequency vibrations on *Xenopus laevis* frog embryos. *Xenopus* embryos exposed to low frequency vibrations (<250 Hz) displayed increased rates of heterotaxia, the randomized placement of visceral organs along the left-right (LR) axis [10]. We found that these patterning defects were due to altered cytoplasmic/cytoskeletal dynamics and tight junctional connections between cells of the early embryo. We also noted that one frequency (15 Hz) additionally produced neural tube defects, suggesting that these two phenotypes (heterotaxia and spina bifida) may have a common intracellular etiology. We thus hypothesized that low frequency vibrations could produce a range of other developmental defects, and could thus be used as a convenient experimental perturbation targeting developmental processes that depend on the cytoskeleton and cell:cell communication.

Here, we have examined the effects of low frequency vibrations on two aquatic species, *Xenopus laevis* and *Danio rerio* (zebrafish). These two species were selected because they have different early embryonic architectures and different types of early cell cleavages, and are both widely used as important laboratory models for understanding environmental contaminants, pattern formation and embryogenesis [5], [7], [11], [12], [13]. Additional advantages are that they are transparent at early stages, allowing for easy scoring of phenotypes; they develop quickly and require relatively little care; and their embryos are available in large numbers allowing for many variables and endpoints to be tested. We focused our analyses on the effects of vibration on LR patterning, neural tube defects, and tail morphogenesis. These endpoints were chosen because prior data suggested that these examples of large-scale pattern formation were especially sensitive to vibration [10], they are early developmental events that are thought to require the cytoskeleton, intracellular communication, and cell-cell contact [14], [15], [16], [17], and they may have common molecular etiologies, as human patients with ZIC3 mutations often have both abnormal LR patterning and neural tube defects [18]. Because vibrations in the environment are likely to vary, we specifically examined the effects of different frequencies, waveforms, and the direction of applied vibration on these endpoints. In Xenopus embryos, we found different effects of vibration frequency, waveform, and applied direction on all three endpoints examined. In zebrafish embryos, we found similar effects of vibration on two endpoints, LR patterning and tail morphogenesis, suggesting that these patterning events may be susceptible to vibrations across a wider range of species.

## Materials and Methods

### Animal Husbandry

Standard protocols were followed for in vitro fertilization of *Xenopus laevis* embryos as described in [19] and embryos were scored according to Nieuwkoop and Faber [20]. Embryos were maintained in 0.1 X Modified Marc’s Ringers (MMR) pH 7.8+0.1% Gentamycin. Standard protocols as described in [21] were followed for collecting fertilized eggs from *Danio rerio* (zebrafish). Zebrafish embryos were maintained in fish water (pH approximately 6.8) with methyline blue (0.00005% final concentration). The studies described in this manuscript were approved by the Animal Care and Use Committee at Tufts University (protocol # M2011-70 and M2011-91) and were conducted according to the NIH Guidelines for the Care and Use for Laboratory Animals. All manipulations performed after neurulation were conducted under treatment with 1.5% MS222 (tricaine), and all efforts were made to minimize suffering.

### Embryo Vibration

Embryos (50–200 per treatment) were placed in approximately 40 ml of 0.1 X MMR in polystyrene petri dishes (Fisherbrand Catalog # 0875712). For treatment with vertical vibrations, the dishes were placed on a 4-inch Sony speaker (Model # 1-544-670-11) connected to a Gwinstek GFG-8216A function generator as described previously [10]. For treatment with horizontal vibrations, the dish was placed on a plexiglass platform attached perpendicular to a 4-inch Sony speaker. The platform was supported by two wheels positioned to run along two horizontal metal tracks, minimizing, but not completely eliminating, vertical vibrations. When activated, the speaker moves the platform horizontally along these tracks.

For *Xenopus* studies, dishes were vibrated in either the horizontal or vertical direction at three frequencies, 7 Hz, 15 Hz and 100 Hz, and three waveforms, sine, triangle and square (Figure S1). These waveforms were selected because of the ability of the frequency generator to produce them; information about which waveform(s) are most environmentally relevant is not currently available. The amplitude of the function generator was kept at the highest non-lethal setting for all experiments. Horizontal and vertical treatments were run in separate rooms so no interference would occur between the machines. The ambient temperature was maintained at ∼22°C and control embryos were maintained in a temperature-matched vibration-free environment. Embryos were vibrated overnight starting at one-cell, with vibrations ending in late neurulation (approximately stage 18–19). After treatment, embryos were kept in incubators ranging from 14–22°C. All embryos were scored at stage 45.

For zebrafish studies, dishes were vibrated in both the horizontal and vertical directions at nine frequencies, 7 Hz, 15 Hz, 20 Hz, 30 Hz, 50 Hz, 70 Hz, 100 Hz, 150 Hz, 200 Hz with sine waveform. These frequencies were chosen because they span the range previously examined in Xenopus [10]. Two frequencies (15 Hz, 150 Hz) were selected to allow further testing of other waveforms (triangle and square waves). Vibrations were applied overnight at ∼22°C and control embryos were maintained in a temperature-matched vibration-free environment. Embryos were vibrated starting at the one- or two-cell stage, and ending at the 5 somite stage. After treatment, embryos were kept in incubators at 28–30°C. All embryos were scored 7 days post fertilization.

### Scoring of Xenopus Phenotypes

All experimental and control tadpoles were scored at approximately stage 45 following treatment with 1.5% MS222 (tricaine) to cease movements. Tadpoles were scored for the presence of three phenotypes: 1) heterotaxia, defined by the abnormal position of the heart, gall bladder, and/or gut loop. 2) Neural tube defects, defined by a dorsalized phenotype with an open neural tube/spinal cord. These animals typically have a tail that splits along the anterior-posterior axis. 3) Bent tail morphology, defined by the presence of distinct bends or kinks anywhere along the spinal column. Tadpoles with bent tails and other minor abnormalities in the head and gut region, such as a narrow head or other craniofacial defect, were included in this category. Tadpoles with a bent tail in addition to other severe defects that affected the whole organism (i.e. edema) and tadpoles with curved spines were not scored as having a bent tail.

### Scoring of Zebrafish Phenotypes

All experimental and control fish were scored following treatment with 1.5% MS222 to cease movements. Fish were scored for the presence of two phenotypes: 1) heterotaxia was assessed using 488/40 nm illumination; at this wavelength, the gut organs autofluoresce. As described previously [22], an embryo was considered heterotaxic if the location of either the pancreas or the gall bladder were on the side opposite normal. Animals with symmetry of these organs (i.e. centrally placed pancreas and gall bladder) were considered examples of isomerisms. 2) Abnormal tail morphology was defined by the presence of distinct bends, kinks or curves anywhere along the spinal column. 3) Neural tube defects were assessed by looking at overt morphology of the spine. Obvious neural tube defects were not observed in any fish regardless of treatment, and more detailed assessments of neural tube closure were not performed.

### Microscopy

Images were taken using a Nikon SMZ1500 dissection microscope with a Retiga camera and ImageQ software. Images were oriented and scaled using GIMP and Adobe Photoshop; data were not added or removed. Original images available upon request.

### Image Analysis

Quantitative analyses of the bent tail phenotype were conducted with ImageJ. Only tails with a single bend, the most common tail phenotype observed, were analyzed. Three measurements were taken from bent tails: the angle of the bend, the distance from the posterior end of the gut coil to the bend in the tail, and the distance from the bend to the tip of the tail. These two distances were combined to determine the total length of the tail. In unaffected tails, the distance from the posterior end of the gut coil to the tip of the tail was measured.

### Statistics

For all treatments, experiments were run in duplicate or triplicate on separate days and pooled. The frequency of each phenotype was calculated as: # of embryos with phenotype/total number of scored embryos. For heterotaxia, the total number included only animals with a normal dorsoanterior index (DAI). A χ^2^ test with Pearson correction for increased stringency was used to compare absolute counts of affected versus unaffected embryos for each treatment and endpoint. Additional phenotypes that were observed occasionally (i.e. hyperpigmentation, edema, etc.) were examined but statistics were not performed for these endpoints. Results were considered significant at p<0.01.

## Results

### 7, 15 and 100 Hz Vibrations Induce Heterotaxia in *Xenopus* Embryos Regardless of Wave Shape or Direction

**Figure 1:**
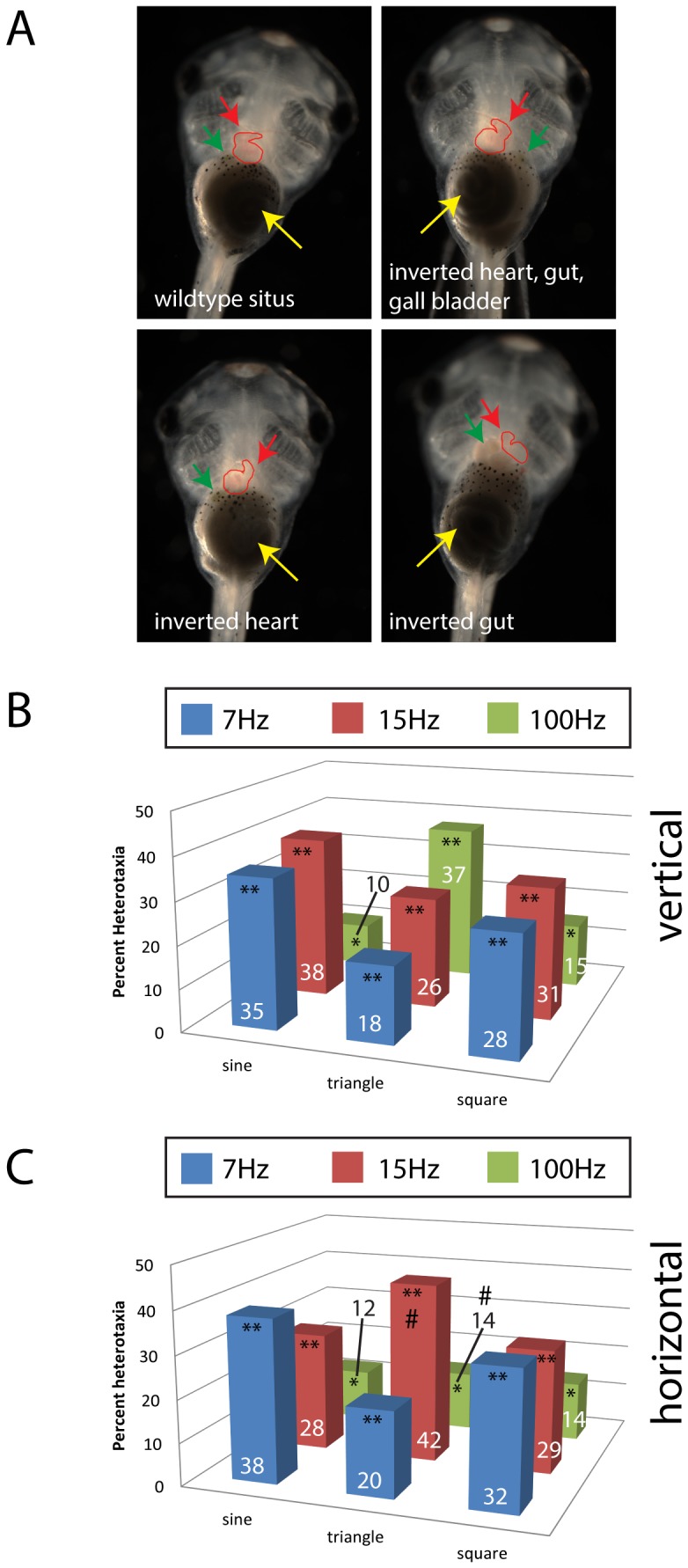
Vibration induces heterotaxia in Xenopus embryos, independent of waveform or direction. A) Shown are embryos with different organ *situs* (position), including three with various forms of heterotaxia, i.e. the inversion in placement of one or more organs. Red arrows indicate the apex of the heart; green arrows indicate the gall bladder; yellow arrows indicate the coiling of the gut. These animals were exposed to one of three vibrations that were previously shown to affect left-right patterning. Here, the waveforms were varied between sine, square and triangle waves. B) Vibrations applied vertically induce heterotaxia, regardless of which frequency and waveform combination was applied. C) Vibrations applied horizontally also induce heterotaxia. Heterotaxia rates in un-vibrated controls were approximately 1%. Numbers on bars indicate frequencies of phenotypes for that treatment. At least 110 embryos were included for each treatment. *p<0.01; **p<<0.001 relative to controls; on panel C, # indicates significant differences from vertical treatment (p<0.01).

In a previous study, vertical sine wave vibrations ranging from 7 to 200 Hz were administered to *Xenopus* embryos, and effects on the orientation of the LR axis were examined [10]. Three frequencies were found to induce significant levels of heterotaxia: 7 Hz, 15 Hz and 100 Hz. To determine the effects of changing the shape of the wave (sine, square, or triangle waveforms) on LR patterning, these three frequencies were administered to Xenopus embryos from 1 cell to stage 19 (late neurulation) and embryos were scored at stage 45 (Figure 1A). All nine treatments induced significant levels of heterotaxia (p<0.001) with varying levels of effectiveness; sine and square waves were most effective at randomizing the LR axis when the administered frequencies were 7 or 15 Hz, but triangle waves were most effective when the administered frequency was 100 Hz (Figure 1B).

Because environmental vibrations are not expected to be limited to vibrations moving in a vertical direction, we next tested whether vibrations applied in the horizontal direction (perpendicular to gravity) would induce alterations in the LR axis. Again, we examined three frequencies (7, 15 and 100 Hz) and three waveforms (sinusoidal, triangular and square waves) and found that all nine treatments significantly induced heterotaxia (Figure 1C). Interestingly, for 7 Hz, sinusoidal and square waves were most effective; for 15 Hz, triangular waves were most effective; and for 100 Hz, there was no apparent influence of wave shape on the magnitude of effect on the LR axis. We conclude that 7 Hz, 15 Hz and 100 Hz vibrations all induce heterotaxia, regardless of the shape of the waveform or the direction the vibrations are applied.

### Vibrations Induce Neural Tube Defects in Xenopus Embryos in a Frequency-, Waveform-, and Direction-specific Manner

**Figure 2:**
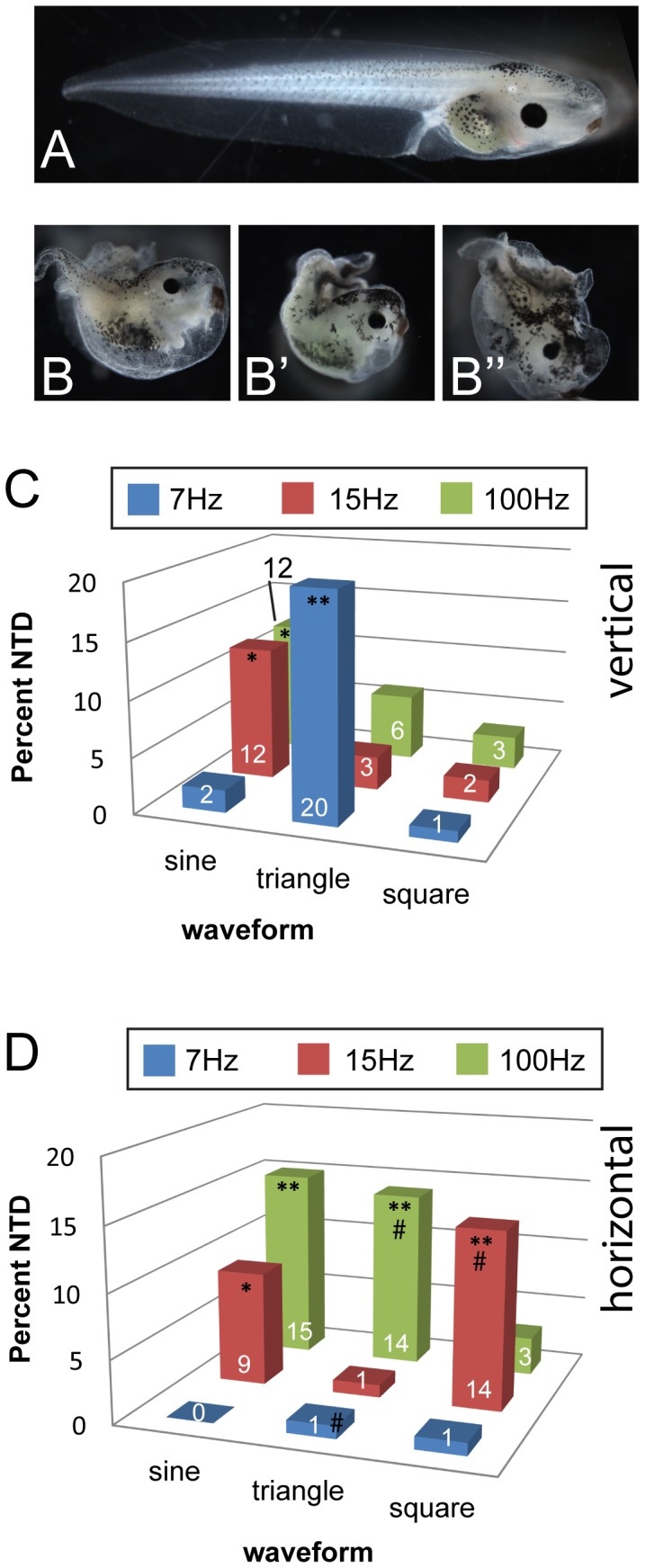
Vibration induces neural tube defects in a frequency-, waveform- and direction-specific manner in Xenopus embryos. *Xenopus laevis* embryos were vibrated from 1-cell through st. 19 (late neurulation) and then allowed to develop in a vibration-free environment approximately stage 45. A) The majority of embryos develop as tadpoles with properly fused spinal cords. B, B’, B”) Examples of tadpoles with neural tube defects (NTDs). NTDs were defined by shortened axes and un-fused spinal cords. Animals often developed split tails. (These animals were produced with 15 Hz sine vertical vibrations.) C) Applying vibrations vertically to embryos induced significant numbers of NTDs at all frequencies tested, but only for certain waveforms. D) NTDs were also observed following vibration in the horizontal direction, but only for specific combinations of frequencies/waveforms. Numbers on bars indicate frequencies of phenotypes for that treatment. NTDs were observed in <1% of controls. Each group includes at least 100 treated embryos. *p<0.01, **p<<0.001 relative to controls; on panel D, # indicates significant differences from vertical treatment (p<0.01).

In our previous study of the effects of low frequency vibrations on the left-right axis, we focused our analyses on vibrations at 7 Hz because we noted that 15 Hz sine vertical vibrations produced neural tube defects in some embryos [10]. Embryos with these defects have incomplete closure of the neural tube which typically manifests as tadpoles with two separate, parallel tail segments (Figure 2), shortened anterior-posterior axes and curved spines; these defects are also often accompanied by malformations in other body structures including craniofacial malformations and edema.

To determine which frequencies, waveforms and directions of applied vibrations produced neural tube defects, we examined embryos following a total of eighteen treatments (resulting from combinations of three frequencies [7 Hz, 15 Hz and 100 Hz], three waveforms [sinusoidal, triangular and square], and two wave directions [horizontal and vertical]). Seven of the eighteen treatments produced significant increases in neural tube defects with frequencies of 6% to 20% compared to just 1% in un-vibrated controls (p<0.01). For vertical vibrations, the most effective treatments were 7 Hz triangle, 15 Hz sine and 100 Hz sine (Figure 2C). For horizontal vibrations, the most effective treatments were 15 Hz sine, 15 Hz square, 100 Hz sine, and 100 Hz triangle (Figure 2D). From these results, we conclude that all there are frequency-, waveform- and wave direction-specific effects on the manifestation of neural tube defects.

### Vibrations Disrupt Morphogenesis of the Tail

**Figure 3:**
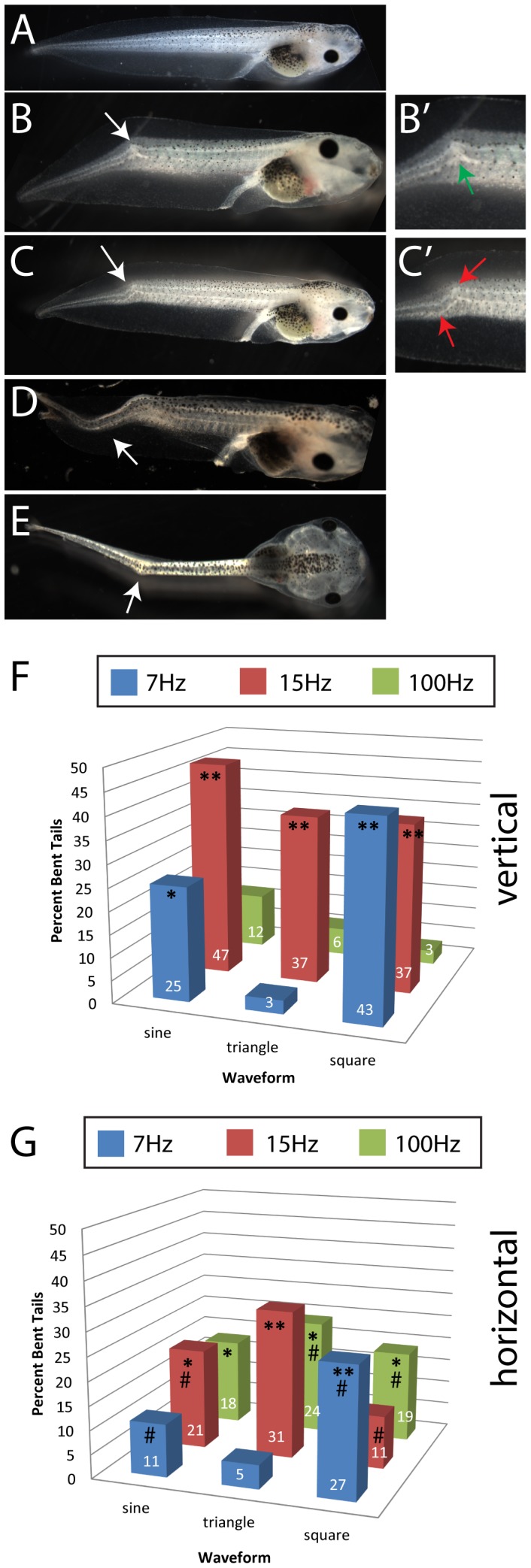
Vibrations induce abnormal tail morphology in Xenopus embryos. *Xenopus laevis* embryos were exposed to vibrations with a range of frequencies, waveforms and directions from 1-cell until approximately st. 19 (late neurulation). These embryos, as well as control embryos raised in a vibration-free environment, were scored at approximately stage 45 for abnormal tail morphologies. Compared to the normal tail appearance (A), a wide variety of bent tail phenotypes were seen, including: B) a single kink in the spine of the tail in the dorsal-ventral plane (kink marked by a green arrow in B’); C) two distinct kinks located within a small distance of each other (both kinks marked by red arrows in C’); D) a more gentle bending of the tail tissue in the dorsal-ventral plane; and E) a distinct kink in the tail in the left-right plane. (Animals shown in panels B-E were all produced with 15 Hz sine vertical vibrations.) For both vertical (F) and horizontal (G) vibrations, several frequencies and waveforms induced significant numbers of tail abnormalities. Numbers on bars indicate frequencies of phenotypes for that treatment. In un-vibrated controls, bent tails were observed in <8%. Each group includes at least 100 tadpoles. *p<0.01, **p<<0.001 relative to controls; on panel G, # indicates significant differences from vertical treatment (p<0.01).

While examining tadpoles for heterotaxia and neural tube defects, we also noted a significant number of treated animals manifesting abnormal tails, with many displaying bent tails starting from approximately stage 43 (Figure 3). In the majority of affected individuals, their tails exhibited a single bend or kink such that the end of the tail bent downward toward the ventral side of the organism (Figure 3B). Whereas most affected tadpoles had only one bend in their tail, some exhibited two or more distinct kinks along the length of this organ (Figure 3C). Additionally, in a number of affected individuals, their tails bent to the left or right side of the body (Figure 3E).

To determine whether vibration frequency, waveform or direction of applied vibrations influenced the incidence of bent tails, we scored the frequency of any bent tail phenotype in the eighteen treatments discussed previously. Importantly, animals with neural tube defects were excluded from these analyses. We found that eleven of the eighteen vibration treatments induced a significant amount of bent tails compared to controls (p<0.01) (Figure 3F [vertical], Figure 3G [horizontal]). Most striking was the incidence of bent tails in horizontally vibrated embryos, with frequencies ranging from 25% to 47% in effective treatments.

To determine whether bent tails had consistent morphological changes, we induced bent tails (with exposure to 15 Hz sine vertical vibrations) and quantified the angle and relative location of the kink in tails with a single bend along the dorsal-ventral axis. These analyses revealed remarkably consistent measures in both the angle and relative location of the tail kink: the bends were, on average, 147.6 degrees (st dev = 7.3 degrees, n = 11) and located 43.9% (st dev = 7.4%) of the way between the end of the gut coil and the tip of the tail. The overall length of bent tails was slightly shorter compared to tails with a normal morphology, but this difference was not significant (bent tails: 6.7±5.3 mm; wildtype tails: 7.0±4.0 mm).

From these results, we conclude that low frequency vibrations alter morphogenesis of the tadpole tail; the effects are frequency-, waveform- and direction-specific. Further, these effects appear to produce relatively consistent effects on the tail, suggesting that vibrations are affecting a particular biological target that is important for tail development. Additionally, these effects are relatively specific, because while some other phenotypes were also observed occasionally (Figure S2), bent tails were observed in large numbers following specific treatments.

### Vibrations Disrupt LR Patterning and Tail Morphogenesis in Zebrafish Embryos

**Figure 4:**
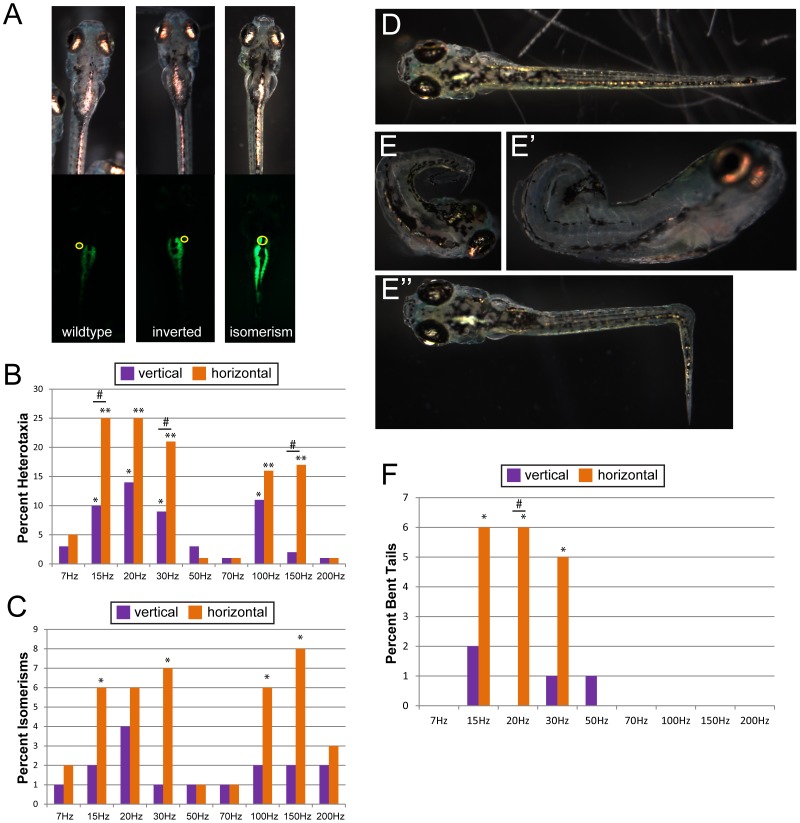
Vibration induces heterotaxia and disrupts tail morphogenesis in Zebrafish embryos. A) Organ *situs* was scored in zebrafish fry 7 days post fertilization using the auto-fluorescence of the pancreas and gall bladder (circled). In vibrated fish, inversions were often observed (with the gall bladder positioned to the left of the pancreas); isomerisms (with the gall bladder directly above the pancreas) were observed less often. These animals were generated by 20 or 30 Hz vertical sine vibrations. B) The incidence of heterotaxia observed in vibrated zebrafish for a total of 18 treatments. Heterotaxia was observed in 3–4% of un-vibrated controls. C) Isomerisms were induced by a few horizontal vibration treatments only. Isomerisms were never observed in untreated fish. D) Typical tail morphology in zebrafish fry at 7 days post fertilization. E-E”) Examples of abnormal tail morphologies observed in zebrafish that were vibrated from 1-cell overnight. These animals were generated by 20 or 30 Hz horizontal sine vibrations. F) Abnormal tail morphologies were rare, but significant numbers were observed following two horizontal vibration treatments. For all graphs, each group includes at least 110 fish. *p<0.01, **p<<0.001 relative to controls; # indicates significant differences between horizontal and vertical treatments (p<0.01).

Although *Xenopus* and zebrafish are both aquatic animals, there are significant differences in the embryonic architecture of these two animals that may influence their susceptibility to interference from vibration treatment. For example, the early Xenopus embryo is mesolecithal, with moderate vegetal yolk disposition and holoblastic (complete) cell cleavages, whereas the zebrafish embryo is telolecithal with dense yolk and a discoidal embryo that sits atop it and undergoes meroblastic (incomplete) cell cleavages. To determine whether vibrations induce alterations in LR patterning, neural tube defects and abnormal tail morphogenesis, an initial screening of nine frequencies applied to zebrafish embryos in two directions (vertical and horizontal) for a total of eighteen treatments were tested. For this first study of zebrafish, only sinusoidal waveforms were examined. We observed heterotaxia following treatment with five frequencies (Figure 4A, 4B); both horizontal and vertical treatments were effective for four of these five frequencies (Figure 4B). Interestingly, four treatments also induced isomerism, or the loss of LR asymmetry, but these were observed only when vibrations were administered horizontally (Figure 4A, 4C).

Bent or other morphologically affected tails were never observed in untreated control fish. However, we did observe bent tails in fish exposed to vibrations (Figure 4D, 4E–E”), including bends along the LR axis as well as curves and bends along the dorsal-ventral axis. Significant numbers of bent tails were observed following two treatments, both of which involved horizontal vibrations (Figure 4F). Importantly, these vibrations are in the range that cannot be administered to *Xenopus* embryos because they produce turbulent movement of the aqueous environment, exposing the embryos to the air-water interface, which is toxic to *Xenopus* but does not affect zebrafish ([10] and data not shown). Neural tube defects were not observed in any fish, regardless of treatment. Taken together, these data suggest that the effects of low frequency vibrations on LR patterning and tail morphogenesis are not species specific, and therefore similar biological/morphological endpoints are affected in aquatic embryos with divergent embryonic architectures.

### Vibrations Affect Patterning of Zebrafish Embryos in a Frequency-, Waveform-, and Direction-specific Manner

**Figure 5:**
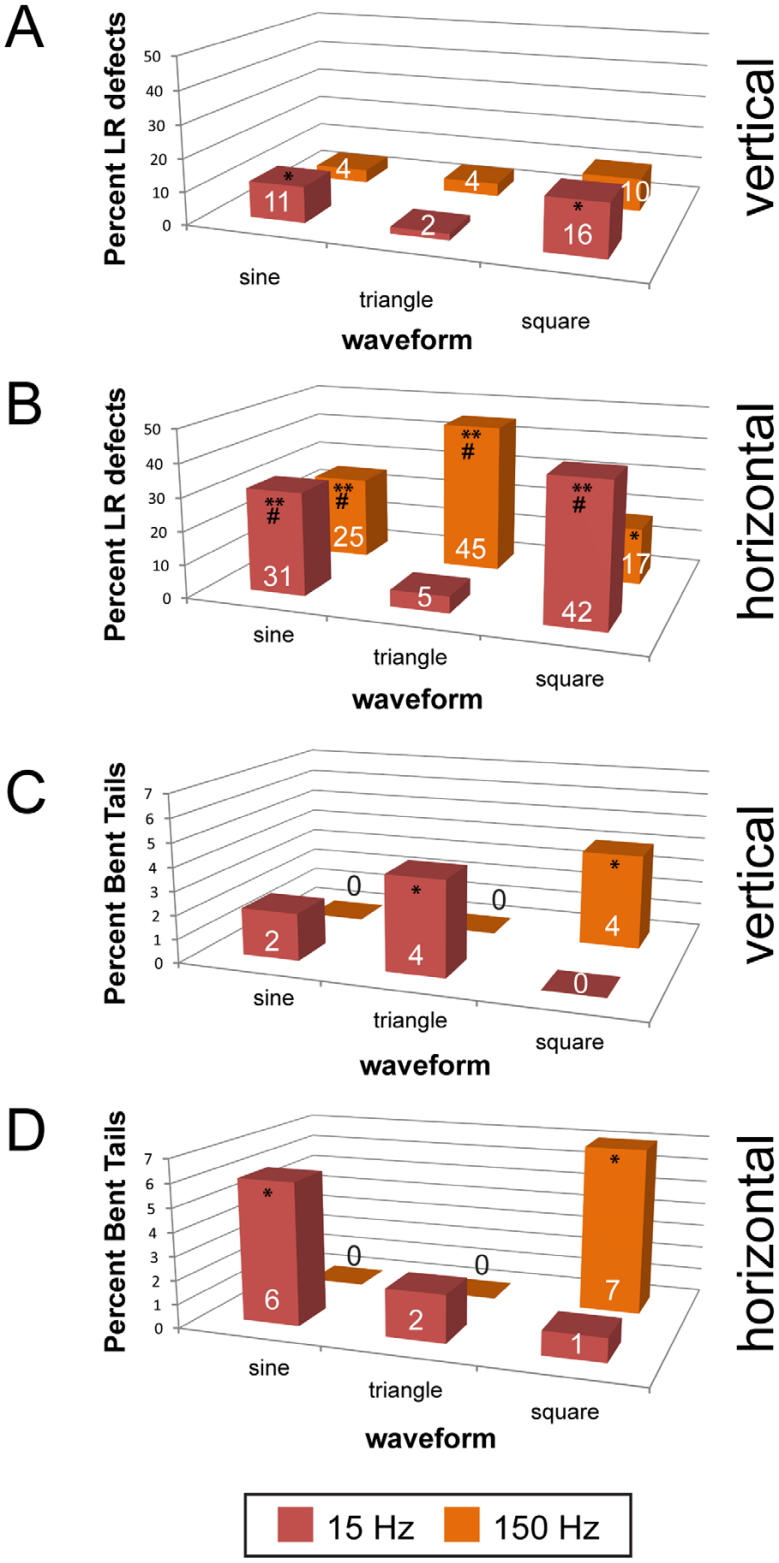
Vibration induces LR patterning and tail morphogenesis defects in zebrafish in a frequency-, waveform- and direction-specific manner. Zebrafish embryos were vibrated from 1-cell through the 5 somite stage, and then allowed to develop in a vibration-free environment until 7 days post-fertilization when they were scored for LR patterning defects (heterotaxia+isomerisms) and abnormal tail morphologies. A) Effects of vibrations applied vertically to LR patterning. Only two treatments induced LR defects. B) Vibrations applied horizontally also induce LR defects. When vibrations were applied in this direction, every treatment with the exception of 15 Hz triangle waves induced significant numbers of LR patterning defects. LR defects were observed in approximately 5% of untreated controls. C) Vertical vibrations induce abnormal tail morphologies in two treatments: 15 Hz triangle and 150 Hz square. D) Horizontal vibrations also induced malformed tails in two different treatments: 15 Hz sine and 150 Hz square waves. Numbers on bars indicate frequencies of phenotypes for that treatment. For all treatments, each group includes at least 110 fish. On graphs, *p<0.01, **p<<0.001 relative to controls; # indicates significant differences between horizontal and vertical treatments (p<0.01).

Our initial screen of 18 treatments identified several frequencies of sine waveform vibrations that disrupt LR patterning and tail morphogenesis in zebrafish embryos. To determine whether zebrafish embryos are affected by other waveforms, we selected two frequencies (15 Hz, 150 Hz) to examine in greater detail. Similar to what we report for Xenopus embryos, we found that zebrafish embryos are more sensitive to some waveforms than to others. When scoring LR patterning defects (heterotaxia+isomerisms), we observed waveform- and direction- specific effects for both 15 Hz and 150 Hz (Figure 5A, 5B). For example, when vertical vibrations were applied, 15 Hz sine and square waves were the only effective treatments (Figure 5A). For horizontally administered waves, every treatment with the exception of 15 Hz triangle waves induced LR patterning defects (Figure 5B).

In addition, several treatments induced bent tails. For vertical vibrations, 15 Hz triangle and 150 Hz square waves induced significant numbers of bent tails (Figure 5C). Following horizontal vibrations, 15 Hz sine and 150 Hz square waves were the most effective. It is also worth noting that the occasional abnormalities we observed in Xenopus (i.e. hyperpigmentation, craniofacial defects, edema, truncated tails; see Figure S2) were never observed in zebrafish, with the exception of edema, which was observed only in a single fish fry (data not shown). From these results, we conclude that low frequency vibrations alter morphogenesis of the zebrafish LR axis and tail morphology in a frequency-, waveform- and direction-specific manner.

## Discussion

We have shown that exposure to low frequency vibrations during early embryonic development causes patterning defects in *Xenopus* and zebrafish embryos, and that the incidences of some of these defects are dependent on specific properties of the vibrational waves. After exposure to vibrations, *Xenopus* embryos developed heterotaxia (Figure 1), neural tube defects (Figure 2), and displayed disruptions in tail morphogenesis (Figure 3). Zebrafish embryos exposed to low frequency vibrations also developed heterotaxia and tail patterning defects (Figure 4, 5), but neural tube defects were not observed. Our results demonstrate that low frequency vibrations are detrimental to early embryonic development in two aquatic vertebrate species and that the characteristics of the vibrations can change the phenotypes that result.

Our results clearly indicate that the incidence of the three phenotypes we scored was significantly affected by vibration parameters (waveform, frequency, and wave direction). In our previous screen of six frequencies (all administered as vertical sine waves), we noted frequency-specific effects on LR patterning in Xenopus [10]. Here, we saw similar frequency-dependent effects on LR patterning in zebrafish, with some frequencies producing high incidences of heterotaxia and others having virtually no effect (Figure 4). Why are some frequencies more effective at altering LR asymmetry, or tail morphogenesis, or neural tube closure than other frequencies? Our previous study indicated that disrupted tight junction integrity and altered cytoplasmic dynamics were responsible for the effects of 7 Hz vibrations on LR patterning [10]. The degree to which vibrations set up standing waves within cytoplasmic structures, and induce movement of intracellular components, are highly dependent on the frequency and wave shape; thus, we hypothesize that only specific frequencies and waveforms, applied parallel or perpendicular to the major embryonic axes, can disrupt the intracellular machinery responsible for these embryonic patterning events. The data we have generated from the testing of different vibration parameters will facilitate future computational modeling of the biological and biophysical processes that are preferentially targeted by each vibration mode.

In several cases, specific treatments produced a specific phenotypic signature. For example, frog embryos that were exposed to 100 Hz vertical triangular waves developed with 35% heterotaxia, 25% bent tails and 5% neural tube defects, whereas frog embryos exposed to 7 Hz horizontal triangular waves developed with 20% heterotaxia, 5% bent tails and no neural tube defects. In both of these cases, these “phenotypic signatures” were different from all other treatments, making them verifiably unique. Thus, the characteristics of an environmental vibration exposure could potentially be determined *a posteriori* by assessing the incidence of three developmental defects. It is therefore plausible that in the environment, the source of vibrational pollution could be identified by comparing the vibration parameters that cause the observed phenotypic signature to the vibrations coming from nearby sources. Our current knowledge about the characteristics of ubiquitous environmental vibrations remains incomplete; although machines and other electronics are known to produce low frequency vibrations, there currently is not enough information to determine what waveform, frequencies and directions are most common, in aquatic or other types of environments.

To date, very little attention has been given to the effects of vibrations on the development of aquatic species. One study examined the effects of various vibrations (20–200 Hz) on three species, axolotl (*Ambystoma mexicanum*), loach (*Misgurnus fossilis*), and guppy (*Lebistes reticulatus*), but no effects on mortality/morbidity or developmental rate were reported [23]. Additional studies have examined the effects of low frequency vibrations on non-aquatic organisms. A recent study shows that low frequency vibrations increase the growth rate and alter metabolic pathways in yeast [24]. In adult rodents, whole-body vibrations (WBV) have been shown to affect bone health, with increased bone formation and decreased osteoclastic activity following 45 Hz treatments for 15 min/day for 3 weeks total [25]. These effects of WBV seem to vary depending on the age of the animal and the density of the applied waves, suggesting that different vibration characteristics can affect health endpoints in a mammalian model as well [26]. Rats exposed to 15 Hz vibrations overnight also demonstrated slight delays in gastric emptying time, an indicator of stress [27], and additional studies of short-term WBV exposures indicate that vibration-induced ulcers develop due to direct effects of vibration and not due to stress responses [28]. In studies examining the effects of vibrations on developing mouse embryos, short-term (10 minute) exposures to vibrations (20 Hz) during embryonic implantation (on embryonic day 4.25) increased the incidence of congenital malformations and reduced birth weights of the surviving pups compared to controls [29]. Similar vibration treatments during early organogenesis (on embryonic day 7) also affected birth weight and body size. Finally, studies of avian embryos indicate that low frequency vibrations induce embryonic death [30], [31], [32], similar to the frequency-specific toxicity we have observed in *Xenopus* embryos (data not shown and [10]). Of the vibrated chicks that hatched, a significant number had congenital defects including missing eyes, crossed beaks, malformed feet and sensory disorientation [31]. Eye and craniofacial defects were observed occasionally in our study in exposed Xenopus embryos (Figure S2), although these were not observed with a high enough incidence to score.

Although experimental studies of vibration *in ovo* and *in utero* did not examine the effects of early exposures, i.e. exposures that correlate to those used in our study where vibrations were applied beginning at the 1-cell stage, they raise an important question: do low frequency vibrations affect human embryonic and fetal development? Embryonic development in aquatic species may be a surrogate to understand similar developmental events in mammals because the mammalian embryo and fetus are localized to an aqueous environment – the amniotic fluid – during development. Furthermore, *Xenopus* and zebrafish are widely acknowledged to be model organisms to understand the effects of environmental pollutants or as developmental models to understand organogenesis, and studies using these animals provide important knowledge to biomedicine [11], [12], [13], [33], [34], [35], [36]. Thus, if vibrations reach the mammalian womb, they may affect the fetus similar to the effects we have observed in aquatic embryos. Previous studies have shown that sound from the external environment does penetrate the womb [37], and that low frequencies (<200 Hz) change very little as they penetrate the uterus and may even increase inside the womb (reviewed in [38]).

In humans, WBV occurs when vibrations get transferred from a weight-supporting surface to the body [39], [40]. Thus, WBV is an occupational hazard [40], [41] that was estimated to affect 6.8 million US workers (reviewed in [40]), with the highest risks seen in operators of industrial machinery including agricultural machines, construction machines, and transportation vehicles including aircraft, trucks, buses, trains and boats [39], [40], [41], [42]. Epidemiology studies suggest that chronic, long-term exposure to WBV is associated with a variety of health problems [39], [40], [41], [42], [43], with risks that increase with duration of exposure [44]. There is also evidence from epidemiology studies that vibrations can affect human development; studies link exposure to vibration during pregnancy with increased rates of spontaneous miscarriages and stillbirths [45] and others indicate that pregnant women exposed to vibrations in certain occupational settings have an increased risk to have children with central nervous system malformations [46]. Although these epidemiology studies are limited in design, they do indicate a plausible link between low frequency vibrations and birth defects in humans.

Our data indicate that low frequency vibrations cause developmental defects in a species-specific manner. Although heterotaxia and abnormal tail morphogenesis were observed in both zebrafish and *Xenopus*, neural tube defects were only observed in Xenopus, and isomerisms were observed only in fish. The use of the frog and fish models allowed us to show that low frequency vibrations negatively affect development across species, but our results indicate that the types of vibration that are the most detrimental may differ between species. From these results, we can speculate that low frequency vibrations may affect mammalian development, but this hypothesis requires further testing.

This study characterized the effects of several vibration variables on three distinct biological endpoints. Future studies of frog and fish embryos should characterize the subcellular, cellular and tissue-based targets of vibration that are responsible for the development of neural tube defects and abnormal tail morphogenesis, as well as the critical periods for the effects of low frequency vibrations on these patterning events. In conclusion, we have shown that low frequency vibrations have toxic effects on developing aquatic species, with frequency- waveform- and wave direction-specific effects on three distinct patterning events: neural tube closure, left-right patterning, and tail morphogenesis. Environmental screening is needed to determine whether wildlife populations are exposed to significant amounts of low frequency vibration. Additional experimental and epidemiology studies will shed light on whether humans are affected by these environmental exposures as well.

## Supporting Information

Figure S1 **Diagram of three waveforms tested in our experiments.** The relative prevalence of each of these modes in various environments remains to be identified.
(TIF)

Figure S2 **Vibration can induce a range of developmental patterning defects in Xenopus embryos.** In addition to scored phenotypes, other severe developmental defects were occasionally observed in treated groups. However, these defects were observed infrequently enough that their incidence was not recorded. A–A”) Edema (indicated by orange arrowheads) was observed in a small number of embryos. This edema was often observed in tadpoles with tail defects including bent and curly tails (indicated by the dotted yellow lines, kinks indicated by green arrows). However, embryos with edemas were not scored for any phenotype due to their severe malformations. B) Hyperpigmentation was occasionally observed, but not related to any specific treatment. C) Craniofacial defects were observed including animals with narrow jaws and conjoined eyes, as shown here (blue arrows). Other craniofacial defects included missing facial structures and malformations in the eyes, mouth, nostrils and otoliths (not shown). D) Occasionally, we observed tadpoles with normal anterior structures but completely truncated tails (red arrowhead).
(TIF)
