# Supplementary figures and images for: Low Frequency Vibrations Induce Malformations in Two Aquatic Species in a Frequency-, Waveform-, and Direction-Specific Manner
Source: PLoS One. 2012 Dec 10;7(12):e51473. doi: 10.1371/journal.pone.0051473 (PMC3519728; doi:10.1371/journal.pone.0051473)

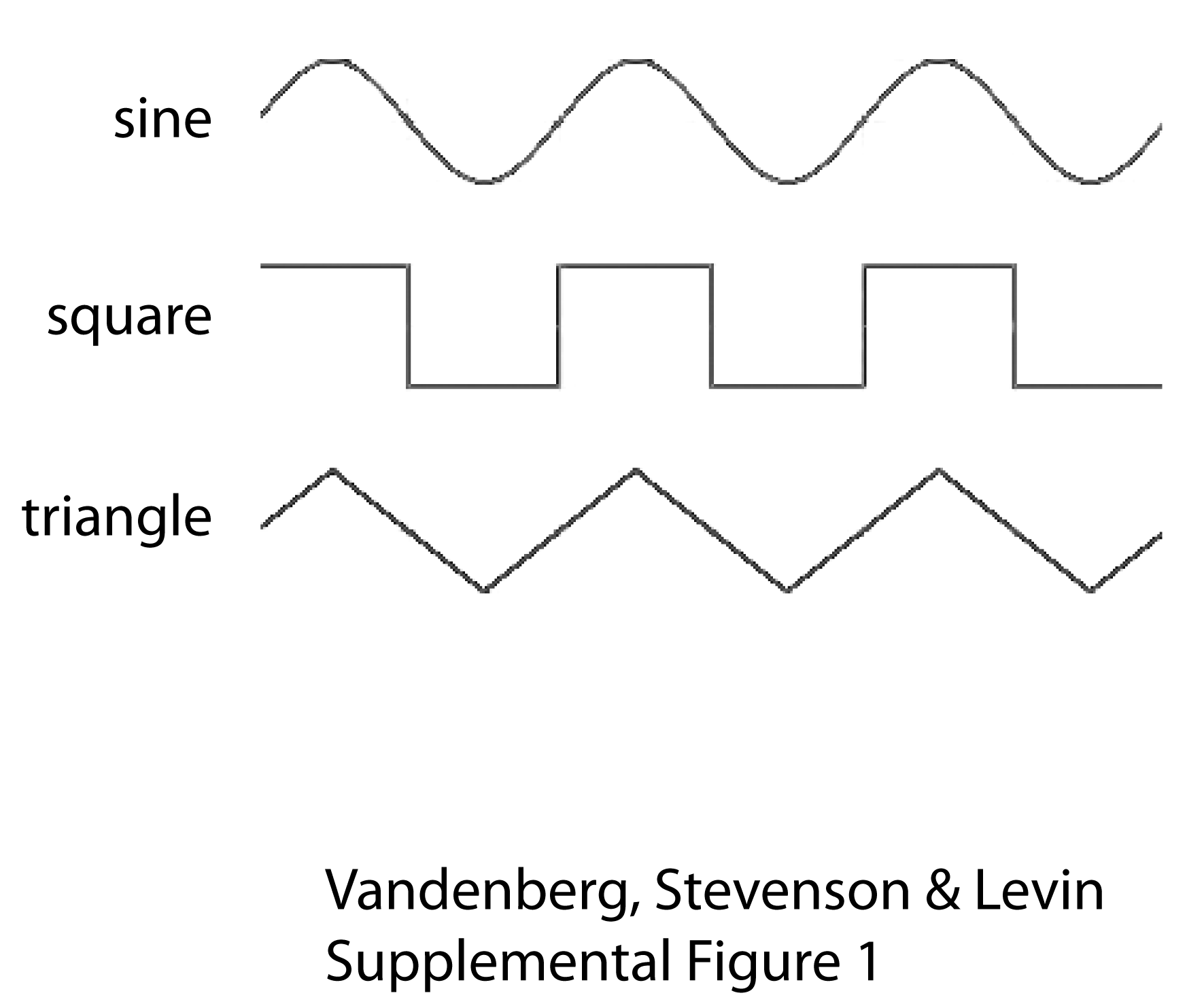

Supplement: Figure S1 — Diagram of three waveforms tested in our experiments. The relative prevalence of each of these modes in various environments remains to be identified. (TIF) [file pone.0051473.s001.tif]

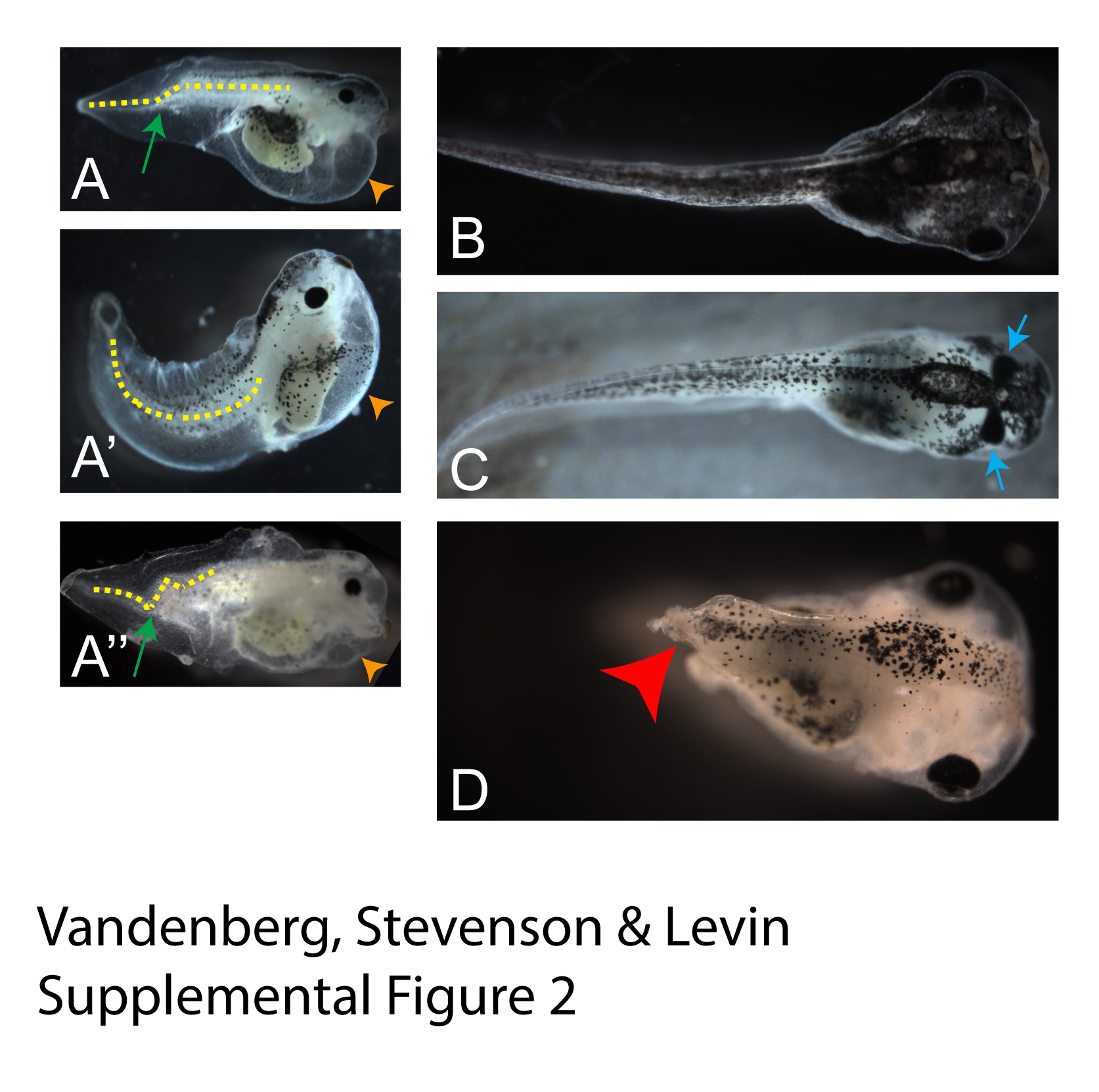

Supplement: Figure S2 — Vibration can induce a range of developmental patterning defects in Xenopus embryos. In addition to scored phenotypes, other severe developmental defects were occasionally observed in treated groups. However, these defects were observed infrequently enough that their incidence was not recorded. A–A”) Edema (indicated by orange arrowheads) was observed in a small number of embryos. This edema was often observed in tadpoles with tail defects including bent and curly tails (indicated by the dotted yellow lines, kinks indicated by green arrows). However, embryos with edemas were not scored for any phenotype due to their severe malformations. B) Hyperpigmentation was occasionally observed, but not related to any specific treatment. C) Craniofacial defects were observed including animals with narrow jaws and conjoined eyes, as shown here (blue arrows). Other craniofacial defects included missing facial structures and malformations in the eyes, mouth, nostrils and otoliths (not shown). D) Occasionally, we observed tadpoles with normal anterior structures but completely truncated tails (red arrowhead). (TIF) [file pone.0051473.s002.tif]
